# Determinants of First-Line Nurse Managers' Span of Control: A Delphi Study

**DOI:** 10.1155/2024/4778460

**Published:** 2024-09-30

**Authors:** Ángel Boned-Galán, Nieves López-Ibort, Ana I. Gil-Lacruz, Ana Gascón-Catalán

**Affiliations:** ^1^ Sterilization Center Miguel Servet University Hospital, Zaragoza, Spain; ^2^ Aragon Health Research Institute (IIS Aragon), Zaragoza, Spain; ^3^ Home Dialysis Unit Lozano Blesa University Clinical Hospital, Zaragoza, Spain; ^4^ Department of Business Management and Organization School of Engineering and Architecture University of Zaragoza, Zaragoza, Spain; ^5^ Department of Physiatry and Nursing Faculty of Health Sciences University of Zaragoza, Zaragoza, Spain

**Keywords:** Delphi method, first-line nurse manager, hospital, management, nursing, span of control

## Abstract

**Aims:** The main goal of this research is to identify, through expert consensus, the key factors that determine the span of control (SOC) of first-line nurse managers (FLNMs) in the Spanish healthcare system.

**Background:** The SOC is a management concept which has usually been defined as the number of subordinates reporting to a superior. In nursing, however, it is much more complex. This complexity is shaped by various factors related to patients, healthcare professionals and organisational structures. Nursing leaders must thoroughly consider these factors and their determinants, which necessitate a comprehensive assessment. Given the significant impact an inadequate SOC can have on patients, professionals and the organisation, it would be beneficial to address this issue. In nursing, studies on this subject are practically nonexistent and focus solely on the number of subordinates, highlighting the need for research in this area.

**Methods:** Between September and December 2022, a Delphi study was conducted. Forty-five experts were invited to participate. The study involved nurse administrators, FLNMs, university professors, renowned researchers and other non–health professionals related to health management. The participants completed an online survey over three phases. Factor analysis was performed on the items for which consensus was reached. The jamovi software version 2.3.15 was used for data analysis.

**Results:** A total of 35 experts participated in at least one of the three phases of the Delphi study. Following a comprehensive analysis of the identified factors, a consensus was reached on 31 of them. These were subsequently grouped into four categories: unit category (16 items, including complexity, resource management, conflicts and protocolisation and monitoring of activities), professional category (five items: number of staff, staffing stability and skill level and diversity of staff), FLNM category (four items: autonomy, experience and education and leadership style) and organisation category (six items: digitisation and information systems, education, research and implementation evidence-based practice and performing guards).

**Conclusions:** Our research shows a high degree of consensus amongst participants in identifying the determinants and degree of relevance of SOC-related aspects. Although SOC is not currently assessed, all stakeholders agree that there are a large number of variables that should be considered when appointing a FLNM.

**Implications for Nurse Managers:** Nursing managers can better assess the health of the organisation and improve performance by understanding the factors that influence the SOC of FLNMs. Due to the lack of previous studies, understanding these factors will allow the development of methods and tools tailored to the characteristics of different health systems.

## 1. Background

Nurse managers play an important role in achieving the goals of healthcare organisations within their respective nursing units and departments [1, 2], especially in aspects relating to leading and coordinating quality improvement work [3]. The nurse manager collective includes first-line nurse managers (FLNMs). This position is defined as individuals who have direct responsibility for patient/resident care units, with nurses and other care providers reporting to them. They are responsible for recruitment and performance management. There is no level of management below them, and they may be responsible for managing more than one unit [4].

One key aspect of the role of FLNMs is human resource management. This is where the concept of span of control (SOC) comes in. SOC originated as a business concept in the 1930s to describe the number of subordinates reporting to a superior [5]. However, the evolution of the term has been nuanced as knowledge of the subject has increased, from the number of full-time equivalent employees [6] to the number of people reporting to a manager [7].

An updated literature review suggests that SOC is a multidimensional phenomenon influenced by a variety of different factors related to the workplace, the unit workers, the FLNM and the organisation [6, 8–12]. Healthcare is becoming complex due to factors such as the need for efficient care, advancements in technology and research, longer life expectancy and the increasing complexity of patient care. In addition, challenges in recruiting and retaining healthcare professionals are contributing to the need for professional practice redesign [13].

SOC theory [14] proposes that there is a size of SOC at which maximum effectiveness is achieved. However, the literature does not offer a specific number or formula for determining the ideal number of direct reports in an optimal SOC [14].

Following this argumentation line, the 2002 Final Report of the Canadian Nursing Advisory Committee encouraged employers to explore and evaluate the characteristics of an appropriate, manageable SOC for clinical managers that allows them to perform assigned functions and be available to meet the needs of nurses and patients [15].

A handful of healthcare-specific studies have examined the impact of SOC on various outcomes: patients (e.g., patient safety and satisfaction) [4, 16, 17], employees (e.g., employee satisfaction, behaviour, turnover and safety) [18–21], FLNM (e.g., influence on leadership, job demands, role, behaviour and skills) [22–24] and organisation (e.g., loss of talent, reputation and organisational outcome) [6, 12, 25].

Only in few health systems, such as the one of Canada [13], a tool has been developed and implemented to determine the SOC of a FLNM. A questionnaire was designed to support objective decision-making on SOC, with a total of eight indicators grouped into three dimensions: staffing, programme and unit characteristics. These dimensions include unit complexity, management of material resources and general services, assigned staff, skills/autonomy (junior professionals), stability (turnover and absenteeism), staff diversity, programme diversity and managed budget.

It should be borne in mind that the health systems of each country are different and condition the functioning of health organisations, so the particular characteristics of each country should be studied in order to propose the most appropriate tool.

Under this background, the perspective of nurses should be considered to identify the main determinants of the SOC of a FLNM in the Spanish health system. To do this, it is necessary to have the vision and experience of experts at the national level.

Amongst the qualitative research techniques, one of the most appropriate for this purpose is the Delphi method. This is a consensus technique developed by the Research and Development Corporation (RAND Corporation) in the 1950s to obtain expert agreement on various phenomena [26]. The Delphi method is now used on the Internet in a form commonly referred to as e-Delphi, where researchers conduct the Delphi in an online survey platform to collect data and facilitate communication between the researcher and experts [27].

The aim of this study is to identify the main factors determining the SOC of FLNMs in hospitals within the Spanish health system, using the Delphi technique and with the participation of a panel of experts on the subject at the national level.

## 2. Methods

### 2.1. Study Design

A three-round Delphi survey (Phases 0, 1 and 2) was implemented, including expert panellists, iterative rounds, statistical analysis and consensus building. The Delphi method was selected to generate ideas from the expert panellists' own knowledge and experience in their different fields of action.

The Delphi method is a technique used to achieve consensus amongst experts from various disciplines on a specific topic [28–31]. Its main strength lies in allowing anonymous interaction amongst experts, who can access the responses of others without knowing their identities or authority on the subject. This enables participants to modify their responses in successive rounds, controlled by a coordinating group, until the responses represent the majority opinion. This study facilitated an anonymous exchange of opinions and expertise, allowing experts to assess and modify their views in line with group consensus, thus promoting a collaborative and informed decision-making process.

The coordinating group consisted of the principal investigator (a nurse with over 24 years of experience in the Spanish public health system, including the last 10 years in diverse management positions) and three experts in management (two specialising in healthcare management and one with a background in business).

The survey was conducted three times via online questionnaires between September and December 2022. LimeSurvey was used as an anonymous online survey tool to send the questionnaires and receive the responses.

### 2.2. Participants

In order to obtain a model valid for the entire Spanish territory and to avoid possible biases due to the characteristics of the regional health services, it was decided to form a panel of expert's representative of the whole country.

The method proposed by Okoli and Pawloski [28] was used to select the panel of experts: (a) nursing administrators, (b) FLNM and (c) other health professionals, e.g., university professors, renowned researchers or other non–health professionals related to health management. A group of 45 national experts was selected and invited to participate through an e-mail describing the objectives and procedures.

### 2.3. Data Collection

#### 2.3.1. Generate an Item Pool

In order not to condition the participants' opinions, they were asked to list aspects related to SOC in Phase 0 of the study. Therefore, this step was explored by asking the expert panellists an open-ended question: ‘List as many key factors as possible to consider in determining the SOC of a FLNM.' There were 30 separate sections within the questionnaire for this purpose, as well as a final section for any additional comments.

#### 2.3.2. Questionnaire Design

Prior to the consultation, a review of the existing literature on the SOC in nursing was undertaken to gain a better understanding of the topic and to identify possible aspects that had not been mentioned by the expert panel.

We implemented a search strategy to identify the existing work related to nurse manager SOC. We searched Medline, Web of Science and Embase for the literature using the following search terms: nurse administrator, nurse manager, FLNM, nursing supervisor, head nurse, nurse management, charge nurse, SOC, span of management, work group size and nursing.

The recommendations of Dillman et al. were followed for the development of the questionnaire and the periodicity of sending reminders for the online questionnaire [29].

The initial questionnaire produced unstructured, qualitative data which were transcribed verbatim into Word documents. A thematic analysis was carried out to identify recurring themes [30, 31]. The documents were systematically examined to identify related or similar themes as well as dissimilar themes. This stage of the analysis was carried out independently by the researcher and the Delphi study coordination group.

Comparative notes were used to validate the emerging concepts. The themes identified were then used to formulate items for the subsequent second and third rounds of questionnaires. These themes formed a first questionnaire, grouped into different dimensions, to be evaluated by the panel of experts. For this purpose, a five-point Likert scale was used to answer the questions, without distinguishing between positive and negative SOC impacts. The questions related to two dimensions: (a) the degree of relevance of the item to the FLNM management SOC (1 = *not relevant*, 2 = *less relevant*, 3 = *relevant*, 4 = *very relevant* and 5 = *extremely relevant*) and (b) whether such an item should be included in a final SOC assessment tool for an FLNM (yes/no). In addition, a space for comments or questions to the coordination group was included.

In the second and third surveys, the response data were tabulated and presented by the researcher, showing the percentage of choices and the distribution of responses for each item. The panellists' comments were used to reformulate or clarify the wording of the various items.

After each phase, the results and relevant comments were anonymised and provided to the participants in a detailed report sent by e-mail.

### 2.4. Extraction of Items: Statistical Analysis

In any Delphi-based study, the definition of consensus must be a priority. Therefore, for this research, consensus was defined using two criteria: (i) more than 80% of the participants in the round should indicate ‘YES' to the need for the item to be part of a tool to determine the SOC of a FLNM, and furthermore, (ii) the item should be rated with an average of three or more on the five-point scale.

jamovi software Version 2.3.15 was used for data analysis. Mode, median, mean, standard deviation, interquartile range and percentages are presented.

### 2.5. Ethical Consideration

Questionnaires were distributed to participants via e-mail accompanied by a link to an anonymous online survey tool (LimeSurvey). Individual names of respondents were anonymised.

The purpose and methods of the study were explained to participants at the beginning of the first questionnaire, and they were informed that their cooperation was voluntary and that there would be no disadvantage for nonparticipation.

Participants' rights to autonomy were respected, and informed consent was obtained through written explanations of the benefits, rights and risks associated with the research study. Consent to participate was implied by the return of the questionnaire, which included an explicit checkbox to accept the conditions of the study. Confidentiality was strictly maintained throughout the data collection phase of the study.

The questionnaires were accessible only to the researcher, which ensured the highest level of confidentiality. Participants shared their opinions anonymously; the data collected were collated to represent group perspectives while maintaining individual anonymity. The results were presented in an aggregate form, representing the collective opinion of the members of the expert panel. This study was approved by the Comité de Ética de la Investigación de la Comunidad Autónoma de Aragón: CEICA (C.P.–C.I. PI22/351).

## 3. Results

The Delphi study was conducted to determine the content/face validity of items that would later be used in the development of a validated assessment instrument.

### 3.1. Experts

**Figure 1:**
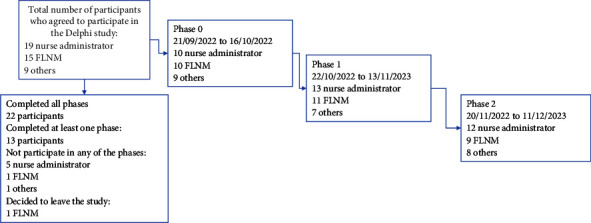
Flowchart of Delphi study phases.

Of the 45 experts invited, 43 accepted the invitation: seven did not participate and one participant decided to leave the study after it had started. The number of participants per phase was 29 in Phase 0, 31 in Phase 1 and 29 in Phase 2. Three phases were completed by 22 participants.  Figure 1 shows the date of completion and details of participation in each phase of the Delphi study. All participants' contributions were taken into account, regardless of whether they participated in one, two or all three phases.

#### 3.1.1. Results of Phase 0 of the Study

A total of 29 experts participated in this first phase of the study by answering the following question: ‘List the most important factors to consider when determining the SOC of a nurse manager.' The number of responses ranged from a maximum of 29 from one participant to a minimum of five, with a mode = 12 and a median = 11 responses.

The answers ranged from very general to very specific, for example, one of the managers gave the following answer ‘The number of professionals who depend on the FLNM', while a FLNM stated the following: ‘The large number of professionals in charge (about 45 amongst the four shifts) of the FLNM, including the time dedicated to calculate personal schedules and adequate coverage of the unit.' The total number of items generated in this phase was 29.

#### 3.1.2. Results of Phases 1 and 2 of the Study

The questionnaire for Phase 1 contained 31 items (29 items from panellists and two items from the literature review) and an open-ended question after each section to collect panellists' questions and comments. The questionnaires for Phase 2 consisted of 34 items, including three items created on the basis of comments from the second survey. A descriptive analysis of the results obtained in Phases 1 and 2 is shown in Table 1.

The table illustrates the evolution in the acceptance of the items by the expert panel, expressed as the mean of the degree of relevance of the item to the FLNM management SOC (1 = not relevant and 5 = extremely relevant). The items related to the unit identified by the expert panel and those added by the coordinating group after consultation of the available literature are shown in Table 2. A total of 15 items were identified in Phase 0 of the Delphi study to be evaluated in Phase 1. In the Phase 1 observations, the expert panel included three new items, which were added to be evaluated in Phase 2. Upon review of the expert panel's comments, it became evident that modifications to the wording or the addition of explanatory texts were necessary to facilitate the comprehension of four items (U04, U05, U11 and U12). Finally, a total of 16 items were agreed in the ‘unit' category, which were grouped into four indicators: complexity (U02, U03, U05, U07, U09, U12, U13 and U15), resource management (U06, U10 and U16), conflicts (U04 and U11) and protocolisation and monitoring of activities (U01, U08 and U14). The Items U17, ‘Interdisciplinary communication and maintenance of care processes and circuits', and U18, ‘The presence of a clinical nurse specialist in the unit to support the management and monitoring of processes and projects', were not accepted.

A total of six items related to professionals were identified in Phase 0 of the Delphi study and were evaluated in Phases 1 and 2 (Table 3). Following the completion of Phase 1, it became necessary to elucidate the meaning of Items P01 and P03. In the case of P01, the following text was added: ‘It will be necessary to assess the need for job-oriented training of the professional (staff of the unit or service, but also “pool” staff).' The only item that was not retained was Item P06, which pertained to the average age of professionals in the unit. Finally, five items were agreed upon in the ‘professionals' category which were grouped into three indicators: the number of staff (P02), staffing stability (P01, P04 and P05) and skill level and diversity of staff (P03).

A total of four items related to FLNMs were identified in Phase 0 of the Delphi study, all of which were evaluated and accepted by the expert panel (Table 4). The category ‘FLNM' was grouped into three indicators: autonomy (F02), experience and education (F03 and F04) and leadership style (F01). Prior to commencing Phase 2, it was observed that Item F03 pertained to a range of training modalities, including those pertaining to master's degrees, soft skills, information technology (IT) and other disciplines.

A total of six items related to organisational characteristics were identified in Phase 0 of the Delphi study, all of which were evaluated and accepted by the expert panel (Table 5). It was necessary to modify the wording of Item O02 and add the following explanatory text: This can be related to new professionals but also to new procedures, techniques or new working dynamics. This process involves the planning, implementation and evaluation of improvement actions. It should be noted that this does not necessarily imply that the entire process must be carried out by the FLNM. The ‘organisation' category was composed of three indicators: digitisation and information systems (O01), education, research and implementation of evidence-based practice (O02, O03, O04 and O05) and performing guards (O06).

## 4. Discussion

To the best of our knowledge, this is the first study carried out on this subject in Spain and in Europe, which could provide the basis for the development of a tool for the objective assessment of the SOC for this geographical context. In Canada, however, there is a study that presents a tool for assessing the SOC, called The Ottawa Hospital Clinical Management SOC (TOH-SOC) decision-making indicator tool [13]. This tool has been used in various hospitals in the United States and Canada, demonstrating the need to assess the SOC and to do so on a regular basis [4, 16, 24, 32, 33].

Comparing the SOC determinants identified by our Delphi study with those used in the TOH-SOC, most of them were identified by our expert panel, and some new ones are included. For instance, the professional's category exhibits a high degree of alignment with the Canadian model, with all items reflected coinciding with those of Canada. Other categories, such as the management of material resources, have been subdivided into a dimension comprising items, thereby differentiating the management of materials, equipment and pharmacy. Amongst those items that do not appear in our research are the following: ‘actual litigation', ‘number of directors' and ‘budget', all of which are more related to the characteristics of the health systems from which the assessment tool originates.

Most studies on the SOC are based on the number of people reporting to a superior and the consequences on the different parties involved. These consequences may include, for example, increased medication errors and nosocomial infections [16], affecting the satisfaction of the FLNM [4] or the organisational behaviours of nurses [23]. Attempting to solve the problem by focussing on the consequences without first identifying the source of the problem may result in inappropriate measures being taken. Consequently, it is imperative to ascertain the factors that influence the SOC of FLNMs in order to implement targeted interventions at the source of the problem.

In general, organisations do not take the SOC into account when designing their staffing structure [34] and, in the case of nursing, it is common to overlap medical units with nursing units, without considering that the role of a FLNM goes far beyond the management of human and materials [19, 32, 35, 36]. It is important to consider the desired outcomes for these units and the organisation, such as improving quality of care, increasing professional satisfaction or reducing costs associated with middle management positions. As resources are limited, managers and organisations need to work together to fine-tune the SOC to achieve the greatest number of objectives [37].

It is at this point that the SOC needs to be measured to assess its adequacy and to provide the necessary support to FLNMs. Understanding the key aspects that determine the SOC can lead to an improved working environment for nurses, the delivery of high quality patient care and a reduction in costs for organisations [38]. In fact, to support FLNMs sometimes is only needed administrative assistance [33] or a comanager [39, 40]. This support can lead to increased job satisfaction and a lower risk of burnout syndrome, which in turn reduces the likelihood of employees leaving their jobs [25, 41]. In addition, it can result in improved performance for the units and teams that they lead.

The results of our study show that 31 items should be considered to determine the SOC of a FLNM. The inclusion of a panel of experts with a variety of perspectives is necessary to get a complete picture of the determinants of the SOC of FLNMs. This study included the views not only of FLNMs, but also of nurse administrators, university nursing professors and health management economists, both active and retired, from different regions of Spain. Although this approach may have reduced consensus on certain points, it enabled us to achieve the proposed objective of this research.

One of the most crucial aspects of implementing any change within an organisational setting is to ensure the involvement of all relevant parties at each stage of the process [42–46]. Only through this approach can critical points that might otherwise go unnoticed be identified. Furthermore, when decisions are made through a shared and consensual process, the implementation and acceptance by employees is more straightforward. This need for diverse perspectives has motivated the inclusion of professionals from different fields in our study. A critical aspect that was taken into account was the positions held by the panellists, who were national nurse leaders. It was recognised that power differentials amongst participants could have a significant impact on the quality of the data if an alternative method, such as focus group interviews, had been used to collect the data.

It is important to note that a large number of the determinants identified relate to the characteristics of the unit or units responsible for the FLNM. These determinants include the ‘unit working environment', which to a large extent influences the interaction between professionals and the degree of support and cooperation [47–51]. If the working environment is positive, it will make it easier to achieve the unit's goals and will allow users to perceive that the staff are working as a team, thereby improving the patient experience. It is noticeable that FLNMs are the ones who rate this item the lowest, especially when they are in direct contact with staff. This may be due to their experience in their own units, with fewer conflicts and a favourable working environment.

In the case of the nurse administrators, they gave greater importance to aspects such as the achievement of objectives and risk management [52, 53], the working climate of the unit [54, 55] and the management style of the FLNM [56, 57]. These results are to be expected, given that their functions and experience give them an overall view of the organisation, a global vision and distance from the aspects that characterise the day-to-day running of the nursing units and services.

On the contrary, the contributions of the FLNMs had their highest scores in aspects related to human resource management, such as staff turnover, number of new professionals and staff absenteeism in their units. These results are not surprising, given that managing staff to keep units running can take up 20%–40% of the FLNM's working time [58]. This can be seen as the visible part of the iceberg of managing a nursing unit.

One aspect that should be highlighted for its novelty, and which is not included in the TOH-SOC tool, is that the panel of experts has added a number of determinants related to the FLNM, such as the management style exercised, the autonomy granted to them in decision-making, training (both before and after access to the management position) and years of experience in management functions. Particularly noteworthy in this study is the training of FLNMs and the low importance they themselves attach to it. In our view, this is one of the fundamental pillars that will characterise their performance as nurse managers. Specific training at the time of taking up the position should not be a prerequisite, but it would be highly recommended that FLNMs receive structured training at the time of taking up the position. Training should be at masters level in management, on the basis that clinical expertise does not prepare the new nurse manager for the wide range of competencies required for success [21, 59]. This will allow them to gain a broader range of knowledge and tools, as well as an overview of the organisation as a whole.

The leadership style employed by FLNMs when directing work teams is of particular relevance. The FLNM is responsible for the leadership of their nursing units, thus becoming a management element with the potential to positively impact the quality of care in their unit. In order for FLNMs to be able to exercise leadership, they must have the necessary conditions in their workplace, including an ideal SOC. In Doran's words, ‘no leadership style can overcome the effects of a wide SOC' [7] in order to achieve these objectives. This training should also be complemented by leadership training, which is essential to strengthen their management skills, promote a positive working environment, improve the quality of care and ensure better coordination and communication within the care team [33, 60]. Of the different types of leadership, FLNMs should be trained in relational leadership [61] and, above all, transformational leadership [22, 62, 63], as it is an effective model for improving healthcare quality and outcomes, professional commitment and patient satisfaction, all through empowering and motivating professionals.

It is interesting to note the assessment of autonomy in decision-making, which was one of the highest rated items overall by the expert panel. As described in previous research, autonomy is associated with higher levels of empowerment and job satisfaction, leading to improved organisational commitment, reduced job stress and higher employee retention [54, 64]. We should not forget that FLNMs, as middle managers in a strategic position between central management and professionals, can provide an operational and efficient response in a variety of situations, thus reducing bureaucracy within organisations. The establishment of this trust in the FLNM's ability to perform its duties effectively represents a model of positive leadership from the nurse administrators towards their subordinates. Furthermore, it demonstrates a commitment to the decentralisation of work and decision-making.

## 5. Limitations

This study has several limitations. Firstly, it was carried out in only one country, Spain. Therefore, the findings are only applicable to the country in which the study was conducted, although they could be relevant to other similar health systems. Furthermore, the outcomes could serve as a foundation for researchers in other contexts or health systems, enabling them to tailor them to their specific circumstances.

Contact with potential members of the expert panel was made through the Asociación Nacional de Enfermeras Gestoras (ANDE), contacts of the coordinating group and the use of social media profiles of other professionals with recognised nursing expertise. This may have resulted in some relevant nurse leaders and their expertise being excluded. In addition, not all experts participated in all phases of the study, so results may have varied depending on their responses.

A disadvantage of the Delphi approach identified in the literature relates to the clear definition of consensus. The literature suggests that 51%–70% agreement represents consensus. In our study, to overcome this limitation and as a strength of the study, consensus was set at over 80%.

## 6. Conclusions

Our research shows a high degree of consensus amongst participants on the determinants and degree of relevance of SOC-related issues. This makes it clear that, although SOC assessment is not currently taken into account, all stakeholders strongly identify the variables that should be taken into account when appointing a FLNM.

The participation of a panel of experts at the national level has allowed the identification of a total of 31 items, grouped into 4 dimensions (unit, professionals, FLNM, and organisation) that should be considered to assess the SOC of FLNMs in Spanish public hospitals.

## 7. Implications for Nursing Management

By understanding the determinants of the SOC of the FLNM, nurse administrators could better understand and assess the status of the organisation and facilitate the FLNM's capacity to adopt an effective leadership style, thereby preventing them from departing from their position. Leadership exerts a considerable influence on the quality of care delivered in their unit and on employee engagement and conduct.

As the results of the present study are refined, it will be possible to develop a tool for weighing the factors influencing SOC, thus facilitating the creation of a common framework for discussion. This tool will enable hospital managements to develop a unified standard for the assessment of the demands of FLNMs, which together with other aspects such as competency development will lead to interventions to improve the working conditions of nurse managers. Creating a robust, grounded tool that objectively assesses SOC will be an important support instrument in assessing the expanding roles of the FLNM. A correct determination of the SOC, carried out periodically, together with the appropriate support measures, can influence an improvement of the results observed in patients, professionals and the organisation.

## Figures and Tables

**Table 1:** Description of items by phases and categories: unit, professionals, FLNM and organisation.

**ID**	**Statement**	**Phase**	**Mean ± SD (1–5)**	**IQR**
U.01	Commitment to risk management and patient safety	1	4.23 ± 0.805	1.0
		2	4.21 ± 0.774	1.0

U.02	Variability/complexity of patient care	1	4.29 ± 0.902	1.0
		2	4.17 ± 0.848	1.0

U.03	Unit operating hours	1	3.26 ± 1.316	1.5
		2	3.41 ± 0.907	1.0

U.04	Unit working climate	1	3.84 ± 1.157	2.0
		2	4.14 ± 1.125	1.0

U.05	Number of different units under the permanent care of the FLNM	1	3.71 ± 0.824	1.0
		2	3.93 ± 1.033	2.0

U.06	Management of a large amount of equipment	1	3.68 ± 1.107	1.0
		2	3.38 ± 1.015	1.0

U.07	Number of patients treated in the unit	1	3.19 ± 1.138	1.5
		2	3.31 ± 1.072	1.0

U.08	Unit monitoring of activities, achievement or service objectives	1	3.90 ± 0.944	2.0
		2	4.07 ± 1.193	2.0

U.09	Interdisciplinary communication and maintenance of care processes and circuits	1	3.45 ± 1.121	1.0
		2	3.59 ± 1.150	1.0

U.10	Management of material resources (not included in automated replenishment systems such as Kanban)	1	3.23 ± 1.023	1.0
		2	3.52 ± 1.184	1.0

U.11	Communication with patients, relatives and/or carers accompanying persons	1	3.10 ± 1.165	2.0
		2	3.62 ± 1.147	2.0

U.12	Patient turnover rate in the inpatient units managed by the FLNM	1	2.48 ± 1.151	1.5
		2	3.21 ± 1.082	1.0

U.13	Frequency with which the capacity of the unit or service is exceeded	1	3.32 ± 1.107	1.0
		2	3.14 ± 1.093	2.0

U.14	Protocolisation, process management and standardisation	1	Na	
		2	3.93 ± 1.033	2.0

U.15	Unpredictability of the work of the unit or service.	1	3.39 ± 1.308	1.0
		2	3.34 ± 1.233	1.0

U.16	Pharmacy management	1	2.90 ± 1.106	2.0
		2	3.07 ± 1.067	2.0

U.17	Having a clinical nurse in the unit to provide support in the management and monitoring of processes and projects	1	Na	
		2	3.31 ± 1.365	2.0

U.18	Level of involvement of the FLNM in the patient admission and/or discharge processes in the care unit	1	Na	
		2	2.66 ± 1.203	1.0

P.01	Number of novice professionals supervised	1	3.74 ± 0.893	1.0
		2	4.14 ± 0.789	1.0

P.02	Number of professionals supervised by the FLNM	1	4.26 ± 0.893	1.0
		2	4.10 ± 0.939	2.0

P.03	Number of different categories of professionals under their responsibility	1	2.84 ± 1.369	2.5
		2	3.45 ± 0.910	1.0

P.04	Absenteeism in the unit	1	2.97 ± 1.278	2.0
		2	3.97 ± 0.865	1.0

P.05	Turnover rate	1	3.58 ± 1.057	1.0
		2	3.93 ± 0.923	0.0

P.06	Average age of professionals in the unit	1	1.71 ± 0.973	1.0
		2	2.41 ± 0.867	1.0

F.01	Leadership style	1	3.65 ± 1.305	2.0
		2	4.14 ± 0.953	1.0

F.02	Decision-making autonomy	1	4.00 ± 1.065	1.5
		2	4.10 ± 1.081	1.0

F.03	Education of the FLNM	1	3.48 ± 1.313	2.0
		2	3.90 ± 0.939	2.0

F.04	Years of experience in management	1	2.23 ± 0.990	2.0
		2	3.10 ± 1.235	1.0

O.01	Digitalisation and technological support: HR management, management of material resources and general services and information systems	1	4.10 ± 1.076	1.0
		2	4.04 ± 0.778	1.0

O.02	Competence assessment and identification of training needs of the unit's professionals	1	3.65 ± 1.199	2.0
		2	4.04 ± 0.906	1.0

O.03	Participation in research projects, EBP, R&D&I and dissemination of results	1	3.00 ± 1.155	2.0
		2	3.82 ± 0.902	1.0

O.04	Presence of students in the unit	1	2.61 ± 0.989	1.0
		2	3.36 ± 0.942	1.0

O.05	Existence of transversal support units to support the FLNM	1	2.74 ± 1.125	1.5
		2	3.32 ± 1.143	2.0

O.06	Carrying out guards as FLNM	1	2.19 ± 1.167	2.0
		2	3.14 ± 1.302	2.0

*Note:* Items not available in Phase 1. The IC of the mean assumes that the sample means follow a *t*-distribution with N-1 degrees of freedom.

Abbreviations: EBP, evidence-based practice; FLNM, first-line nurse manager; IQR, interquartile range; Na, not available; O, organisation; P, professional; R&D&I, research, development and innovation; U, unit.

**Table 2:** Rating of items on unit characteristics by phase, expert group and overall agreement percentage.

**Unit characteristics**		**Delphi Phase 1**			**Delphi Phase 2**			**Was the item accepted?**
**ID**	**Statement**	**Group**	**Mean**	**Item acceptance**	**Group**	**Mean**	**Item acceptance**	
U.01	Commitment to risk management and patient safety	NA	4.31	96.77%	NA	4.58	100%	Yes
		FLNM	4.09		FLNM	4.00		
		Others	4.29		Others	3.88		

U.02	Variability/complexity of patient care	NA	4.31	96.77%	NA	3.92	100%	Yes
		FLNM	4.00		FLNM	4.11		
		Others	4.71		Others	4.63		

U.03	Unit operating hours	NA	3.31	87.10%	NA	3.83	100%	Yes
		FLNM	3.27		FLNM	3.00		
		Others	3.14		Others	3.25		

U.04	Unit working climate	NA	4.08	90.32%	NA	4.50	96.55%	Yes
		FLNM	3.45		FLNM	3.22		
		Others	4.00		Others	4.63		

U.05	Number of different units under the permanent care of the FLNM	NA	3.46	100%	NA	3.58	96.55%	Yes
		FLNM	4.00		FLNM	4.33		
		Others	3.71		Others	4.00		

U.06	Management of a large amount of equipment	NA	3.69	93.55%	NA	3.08	96.55%	Yes
		FLNM	3.55		FLNM	3.78		
		Others	3.86		Others	3.38		

U.07	Number of patients treated in the unit	NA	3.38	77.42%	NA	3.00	96.55%	Yes
		FLNM	2.82		FLNM	3.11		
		Others	3.43		Others	4.00		

U.08	Unit monitoring of activities, achievement or service objectives	NA	4.15	100%	NA	4.58	93.1%	Yes
		FLNM	3.36		FLNM	3.44		
		Others	4.29		Others	4.00		

U.09	Interdisciplinary communication and maintenance of care processes and circuits	NA	3.62	87.10%	NA	3.92	93.1%	Yes
		FLNM	3.18		FLNM	3.00		
		Others	3.57		Others	3.75		

U.10	Management of material resources (not included in automated replenishment systems such as Kanban)	NA	3.15	96.77%	NA	3.50	93.1%	Yes
		FLNM	3.36		FLNM	3.56		
		Others	3.14		Others	3.50		

U.11	Communication with patients, relatives and/or carers accompanying persons	NA	3.15	80.64%	NA	3.92	93.1%	Yes
		FLNM	3.00		FLNM	2.89		
		Others	3.14		Others	4.00		

U.12	Patient turnover in the inpatient units managed by the FLNM	NA	2.46	64.52%	NA	3.00	93.1%	Yes
		FLNM	2.27		FLNM	3.11		
		Others	2.86		Others	3.63		

U.13	Frequency with which the capacity of the unit or service is exceeded	NA	2.77	80.64%	NA	3.17	93.1%	Yes
		FLNM	4.00		FLNM	2.89		
		Others	3.29		Others	3.38		

U.14	Protocolisation, process management and standardisation	Items added in Phase 2			NA	4.00	93.1%	Yes
					FLNM	4.00		
					Others	3.75		

U.15	Unpredictability of the work of the unit or service	NA	3.62	83.87%	NA	3.50	82.75%	Yes
		FLNM	3.64		FLNM	3.33		
		Others	2.57		Others	3.13		

U.16	Pharmacy management	NA	2.92	87.10%	NA	3.17	82.75%	Yes
		FLNM	2.64		FLNM	3.11		
		Others	3.29		Others	2.88		

U.17	Having a clinical nurse in the unit to provide support in the management and monitoring of processes and projects	Item added in Phase 2			NA	3.33	75.86%	No
					FLNM	3.67		
					Others	2.88		

U.18	Level of involvement of the FLNM in the patient admission and/or discharge processes in the care unit	Items added in Phase 2			NA	2.42	65.51%	No
					FLNM	2.89		
					Others	2.75		

*Note:* Items accepted: More than 80% of participants agreed with the item, with an average score of three or more on the five-point scale.

Abbreviations: FLNM, first-line nurse manager; NA, nurse administrator.

**Table 3:** Rating of items on professional characteristics by phase, expert group and item acceptance.

**Characteristics of professionals**		**Delphi Phase 1**			**Delphi Phase 2**			**Was the item accepted?**
**ID**	**Statement**	**Group**	**Mean**	**Item acceptance**	**Group**	**Mean**	**Item acceptance**	
P.01	Number of novice professionals supervised	NA	3.69	93.55%	NA	3.92	100%	Yes
		FLNM	3.91		FLNM	4.33		
		Others	3.57		Others	4.25		

P.02	Number of professionals supervised by the FLNM	NA	4.15	100%	NA	3.83	100%	Yes
		FLNM	4.64		FLNM	4.00		
		Others	3.86		Others	3.75		

P.03	Number of different categories of professionals under their responsibility	NA	2.85	74.19%	NA	3.33	100%	Yes
		FLNM	2.64		FLNM	3.44		
		Others	3.14		Others	3.63		

P.04	Absenteeism in the unit	NA	3.00	74.19%	NA	3.67	96.55%	Yes
		FLNM	2.73		FLNM	4.33		
		Others	3.29		Others	4.00		

P.05	Turnover rate	NA	3.85	87.10%	NA	3.83	93.10%	Yes
		FLNM	3.18		FLNM	4.53		
		Others	3.71		Others	4.25		

P.06	Average age of professionals in the unit	NA	1.85	32.26%	NA	2.75	65.52%	No
		FLNM	1.45		FLNM	2.00		
		Others	1.86		Others	2.38		

*Note:* Items accepted: More than 80% of participants agreed with the item, with an average score of three or more on the five-point scale.

Abbreviations: FLNM, first-line nurse manager; NA, nurse administrator.

**Table 4:** Rating of items on FLNM characteristics by phase, expert group and item acceptance.

**FLNM characteristics**		**Delphi Phase 1**			**Delphi Phase 2**			**Was the item accepted?**
**ID**	**Statement**	**Group**	**Mean**	**Item acceptance**	**Group**	**Mean**	**Item acceptance**	
F.01	Leadership style	NA	3.77	83.87%	NA	4.33	96.55%	Yes
		FLNM	3.27		FLNM	3.67		
		Others	4.00		Others	4.38		

F.02	Decision-making autonomy	NA	3.92	90.32%	NA	3.92	93.1%	Yes
		FLNM	4.00		FLNM	4.00		
		Others	4.14		Others	4.50		

F.03	Education of the FLNM	NA	3.92	87.10%	NA	4.08	93.1%	Yes
		FLNM	2.64		FLNM	3.67		
		Others	4.00		Others	3.77		

F.04	Years of experience in management	NA	2.23	51.61%	NA	3.17	82.75%	Yes
		FLNM	2.27		FLNM	2.78		
		Others	2.14		Others	3.38		

*Note:* Items accepted: More than 80% of participants agreed with the item, with an average score of three or more on the five-point scale.

Abbreviations: FLNM, first-line nurse manager; NA, nurse administrator.

**Table 5:** Rating of the items on the organisation characteristics, by phases, expert group and item acceptance.

**Characteristics of organisations**		**Delphi Phase 1**			**Delphi Phase 2**			**Was the item accepted?**
**ID**	**Statement**	**Group**	**Mean**	**Item acceptance**	**Group**	**Mean**	**Item acceptance**	
O.01	Digitalisation and technological support: HR management, management of material resources and general services and information systems	NA	4.23	96.77%	NA	3.91	100%	Yes
		FLNM	3.91		FLNM	4.22		
		Others	4.14		Others	4.00		

O.02	Competence assessment and identification of training needs of the unit's professionals	NA	3.23	87.10%	NA	3.82	100%	Yes
		FLNM	4.00		FLNM	4.56		
		Others	3.86		Others	3.75		

O.03	Participation in research projects, EBP, R&D&I and dissemination of results	NA	3.54	80.64%	NA	3.73	96.55%	Yes
		FLNM	2.27		FLNM	4.11		
		Others	3.14		Others	3.63		

O.04	Presence of students in the unit	NA	2.77	80.64%	NA	3.18	96.55%	Yes
		FLNM	2.45		FLNM	3.56		
		Others	2.57		Others	3.38		

O.05	Existence of transversal support units to support the FLNM	NA	3.08	67.74%	NA	3.27	96.55%	Yes
		FLNM	2.36		FLNM	3.11		
		Others	2.71		Others	3.63		

O.06	Carrying out guards as FLNM	NA	2.08	51.61%	NA	3.55	82.75%	Yes
		FLNM	2.18		FLNM	3.22		
		Others	2.43		Others	2.50		

*Note:* Items accepted: More than 80% of participants agreed with the item, with an average score of three or more on the five-point scale.

Abbreviations: EBP, evidence-based practice, FLNM, first-line nurse manager; NA, nurse administrator; R&D&I: research, development and innovation.

## Data Availability

The data that support the findings of this study are available from the corresponding author upon reasonable request.
